# Toward utilization of data for program management and evaluation: quality assessment of five years of health management information system data in Rwanda

**DOI:** 10.3402/gha.v7.25829

**Published:** 2014-11-19

**Authors:** Marie Paul Nisingizwe, Hari S. Iyer, Modeste Gashayija, Lisa R. Hirschhorn, Cheryl Amoroso, Randy Wilson, Eric Rubyutsa, Eric Gaju, Paulin Basinga, Andrew Muhire, Agnès Binagwaho, Bethany Hedt-Gauthier

**Affiliations:** 1 Research Department, Partners In Health/Inshuti Mu Buzima, Kigali, Rwanda; 2 Division of Global Health Equity, Brigham and Women's Hospital, Boston, MA, USA; 3 HMIS Department, Ministry of Health, Kigali, Rwanda; 4 Partners In Health, Boston, MA, USA; 5 Department of Global Health and Social Medicine, Harvard Medical School, Boston, MA, USA; 6 Integrated Health Systems Support Project, Management Sciences for Health, Kigali, Rwanda; 7 Integrated Delivery, Bill and Melinda Gates Foundation, Seattle, WA, USA; 8 Geisel School of Medicine, Dartmouth College, Hanover, NH, USA; 9 School of Public Health, College of Medicine and Health Sciences, University of Rwanda, Kigali, Rwanda

**Keywords:** health management information system, global health, data quality, quality improvement, data use, Rwanda

## Abstract

**Background:**

Health data can be useful for effective service delivery, decision making, and evaluating existing programs in order to maintain high quality of healthcare. Studies have shown variability in data quality from national health management information systems (HMISs) in sub-Saharan Africa which threatens utility of these data as a tool to improve health systems. The purpose of this study is to assess the quality of Rwanda's HMIS data over a 5-year period.

**Methods:**

The World Health Organization (WHO) data quality report card framework was used to assess the quality of HMIS data captured from 2008 to 2012 and is a census of all 495 publicly funded health facilities in Rwanda. Factors assessed included completeness and internal consistency of 10 indicators selected based on WHO recommendations and priority areas for the Rwanda national health sector. Completeness was measured as percentage of non-missing reports. Consistency was measured as the absence of extreme outliers, internal consistency between related indicators, and consistency of indicators over time. These assessments were done at the district and national level.

**Results:**

Nationally, the average monthly district reporting completeness rate was 98% across 10 key indicators from 2008 to 2012. Completeness of indicator data increased over time: 2008, 88%; 2009, 91%; 2010, 89%; 2011, 90%; and 2012, 95% (*p*<0.0001). Comparing 2011 and 2012 health events to the mean of the three preceding years, service output increased from 3% (2011) to 9% (2012). Eighty-three percent of districts reported ratios between related indicators (ANC/DTP1, DTP1/DTP3) consistent with HMIS national ratios.

**Conclusion and policy implications:**

Our findings suggest that HMIS data quality in Rwanda has been improving over time. We recommend maintaining these assessments to identify remaining gaps in data quality and that results are shared publicly to support increased use of HMIS data.

National health data are required for planning and evaluation of service delivery (1–3) . This planning and evaluation is critical in developing countries where the majority of health services are provided through national programs and the limited funds must be used efficiently and effectively (1–4) . In these settings, high data quality is important to ensure that decisions reflect program needs and direct health professional education priorities (2–6) . Poor data quality not only contributes to poor decisions and loss of confidence in the systems, but also threatens the validity of impact evaluation studies (7).

In most countries, health management information systems (HMISs) serve as the primary data source for national health planning and evaluation (2, 4). However, existing evidence suggests variable and often poor quality of this data (7–15) . In 2009, the World Health Organization (WHO) shared a framework for assessing data quality of HMIS through checks of completeness, internal consistency and external consistency (16), offering countries a way to measure data quality and identify gaps.

The Rwanda Ministry of Health (MoH) introduced an electronic-based HMIS in 2008. Given an established electronic system, there is an opportunity to use HMIS data for evaluation purposes and policy making in Rwanda. It can also provide national-level estimates as representative surveys are expensive and can only be done after 3–5 years, and they do not necessarily provide estimates at the lowest catchment area of service delivery (17). While examples exist of interventions conducted in Rwanda to improve HMIS data quality (18–20) , no formal assessment of quality of Rwanda HMIS data exists. The purpose of this study is to assess the quality of the Rwanda HMIS data from 2008 to 2012.

## Methods

### Rwanda National HMIS

Prior to 2008, the Rwanda HMIS existed almost entirely in paper form. Rwanda began using an electronic HMIS in 2008 to capture facility healthcare data. Indicators collected include service uptake data for key programs (e.g. immunization, family planning, and antenatal care) and general health systems data (e.g. drug availability and financial information). Patient-level data are recorded in paper-based registers by care providers. Data are aggregated at the facility-level and monthly reports are submitted to the district team. Prior to 2012, reports were then forwarded to the central MoH office and imported into an electronic system. Since 2012, MoH introduced a web-based system (DHIS2) allowing data entry to be done at the facility. This system allows data to be stored centrally, and the facility to maintain and view their data from a local database. In 2012, there were 922 health facilities in Rwanda, 748 (81%) of which were public. The remaining 174 (19%) were private.

### WHO data quality report card

Noting the importance of HMIS data with regards to national and sub-national health sector planning, the WHO introduced the data quality report card framework (16). This framework provides standardized methods for assessing data quality in different low-income settings around the world, and outlines a series of checks that can be conducted quickly to identify inconsistencies in national HMIS systems.

### Data and analysis

Data were extracted from Rwanda's national HMIS database covering all facility reports from January 2008 through December 2012. Using the WHO report card framework (16), we assessed the data quality of the 495 publicly funded health facilities that were open for the duration of the reporting period. The assessment focused on two dimensions of quality: completeness and internal consistency of reported data. Ten indicators were included in the assessment, selected based on WHO recommendations and priority areas for the national health sector (Table 1).

**Table 1:** List of indicators included in the HMIS data quality

		Indicators
I1	ANC1	New ANC registration
I2	ANC4	Women who completed four ANC standard visits
I3	OPD	Outpatient visits
I4	Deliveries	Total deliveries
I5	FP	Women who used family planning at the end of the month
I6	Riskrefer	Number of patients referred to hospitals
I7	DTP1	Children who received diphtheria–pertussis–tetanus first dose
I8	DTP2	Children who received diphtheria–pertussis–tetanus second dose
I9	DTP3	Children who received diphtheria–pertussis–tetanus third dose
I10	U5visit	Number of under the age of five children visits

### Completeness of reported data

Completeness of reporting at health facility and completeness of indicator data in a report were measured on indicators 1–10 (Table 1).

#### Completeness of facility reporting

At the national level, completeness of facility reporting was measured as the number of monthly reports received divided by the expected number of reports in a given year (12×number of health facilities reporting that year). At district level, the proportion of districts that have facility reporting rates below 80% was calculated. These districts are considered to have poor reporting.

#### Completeness of indicator data

Completeness of indicator data was measured as percentage of values that are not missing values for key indicators. At the national level, this percentage is calculated by summing all the non-missing values across key indicators for a specified period of time and dividing by the expected number (12 months×30 districts×10 indicators). A district was considered to have incomplete indicator reporting if it reported more than 20% of missing values across 10 indicators.

### Internal consistency of reported data

Extreme and moderate outliers for indicators 1–10, trends over time for indicators 1, 3, 4, and 9, and internal consistency of I7 (compared to I1) and I9 (compared to I7) were examined.

#### Moderate and extreme outliers

Moderate outliers were defined as monthly values that were at least ±2 standard deviations from the average value of the indicator for a given district for a specified period of time. Extreme outliers were at least ±3 standard deviations.

#### Internal consistency between indicators

Consistency between new Antenatal Care registration (ANC1) and Diphtheria–Pertussis–Tetanus first dose (DTP1) was measured by calculating a DTP1/ANC1 ratio for each district. These ratios were recommended by the WHO framework because the indicators in each ratio are expected to track one another. If the district ratio was 33% different from the national ratio, it was considered to be inconsistent. Consistency between DTP1 and Diphtheria–Pertussis–Tetanus third dose (DTP3) was calculated by dividing total number of DTP3 by the total number of DTP1 for each district. Percentage of districts that have DTP3 immunizations number that are 2% or higher than DTP1 which is a marker of inconsistent were reported.

#### Consistency over time

The check for consistency over time calculated the ratio of the reported values in 2011 and 2012 for a specific indicator to the mean value of the same indicator for the previous 3 years combined. At the subnational level, this indicator looks at the percentage of districts with at least 33% difference between their ratio and the national ratio, a marker of inconsistency.

## Results

Completeness of facility reporting increased from 2008 to 2012 (Table 2). Seven percent of districts in 2008 reported a completeness rate below 80%, which decreased to 0% in 2012. Completeness of indicator data increased over time from 88% in 2008 to 95% in 2012 (*p*<0.0001). The proportion of districts with >20% missing values decreased from 7% in 2008 to 0% in 2012.

**Table 2:** Completeness of facility reporting and indicator data (2008–2012)

	2008 (%)	2009 (%)	2010 (%)	2011 (%)	2012 (%)
National district completeness rate	95	99	98	100	100
Districts with completeness rate below 80%	7	0	0	0	0
Completeness of indicator data	88	91	89	90	95
Proportion of district with more than 20% missing values	7	0	3	3	0

At the national level, the percentage of moderate and extreme outliers was 0% across all years (Table 3). At the sub-national level, no districts reported >5% monthly values that were extreme or moderate outliers. At the facility level, the mean percent of outliers was 4% (2008, 2009, 2010 and 2011) and 3% in 2012. Extreme outliers were found only in 2012 (3%). In 2008, 10% of districts had DTP1/ANC1 ratios above the national ratio. This percentage decreased to 0% in 2012. In 2009, 13% of districts had DTP1/ANC1 ratio below the national ratio, which decreased to 0% in 2012. The percentage of districts where the DTP3/DTP1 ratio was >2% was high in 2009 (17%) and 2012 (23%).

**Table 3:** Outliers and internal consistency between indicators (2008–2012)

	2008	2009	2010	2011	2012
Extreme and moderate outliers					
Proportion of values that are moderate outliers^a^	0%	0%	0%	0%	0%
Proportion of values that are extreme outliers^a^	0%	0%	0%	0%	0%
Internal consistency between DTP1 and ANC1					
National DTP1/ANC1 ratio	0.87	0.97	0.87	0.90	0.94
Proportion of districts with DTP1/ANC1 ratio 33% above national ratio	10%	%	0%	0%	0%
Proportion of districts with DTP1/ANC1 ratio 33% below national ratio	0%	13%	0%	0%	0%
Internal consistency between DTP1 and DTP3					
National DPT3/DTP1 ratio	0.96	1.00	1.01	0.97	0.99
Proportion of districts where DTP3 is 2% greater than DTP1	13%	17%	3%	0%	23%

a Numerator=sum of occurrences of outliers [±2 (3) SD] over the 12 months for the 10 indicators; Denominator=120 (number of health facilities×12 months×10 indicators).

Table 4 shows the consistency over time ratios for 2011 and 2012. There was a 21% increase in reported deliveries in 2011 compared to the mean of three preceding years, with a 14% increase in 2012. For the outpatient department visit ratio, there was a 10% decrease in 2011 and a 13% increase in 2012. For all other indicators, the change was minimal.

**Table 4:** Consistency over time: national ratio of total number of events in the current year to mean number of events in preceding 3 years

	2011	2012
ANC1 ratio	1.02	1.02
Deliveries ratio	1.21	1.14
DTP3 ratio	1.00	1.05
OPD ratio	0.90	1.13
Proportion of district with 33% difference between their ratio and national ratio	0%	10%

## Discussion

Overall, our data quality assessment suggests high and increasing completeness of reporting and internal consistency of the Rwanda HMIS data. The improvement is likely attributable to interventions implemented in the country by the Rwandan government and non-government organizations to strengthen health systems and improve data quality. Performance Based Financing (PBF) (21), introduced in 2010, is one such intervention that may have contributed to improved data quality Since HMIS reports provide data that guided incentives payments for PBF, the MoH established rigorous quality checks of the HMIS data by district supervisors as part of their formative monthly supervision (19, 20, 22). Change in technology from locally based system to a web based system, and trainings on how to use the system and data cleaning done at health facility have also highly contributed to this improvement. This is important because Rwanda's HMIS data is a data source for local, national and international policy-makers and demonstrating high data quality may encourage the use of this data more broadly (17).

While we found improvement in completeness, other metrics identified potential data challenges. We found deviations in the consistency over time measures for deliveries and OPD visits. While these findings may indicate poor data quality, they could be explained by increased uptake of services (21–23) . An increased DPT3/DPT1 ratio could result from migration within a district where the number of children eligible for DPT3 increases or decreases or if more vaccines were given at the beginning or end of a year.

Our results contrast with the other published assessment of HMIS using the WHO report card framework in sub-Saharan Africa, where they found poor data quality (24). They also differ with most results of different assesments of facility data quality, which also found gaps in data quality pointing to a need for improvement (7–15) . Another study from Mozambique, using a Global Fund methodology, also found high quality for assessed indicators (3).

Our analysis has limitations. First, private health facilities were excluded. In 2012, private facilities accounted for 19% of all facilities in Rwanda and accounted for an estimated 11–15% of health service delivery (A. Muhire, personal communication, October 16, 2014). Private facilities only started reporting to HMIS in 2012, and due to the difference in implementation time between public and private facilities, we felt they should be analyzed separately. Second, although chosen *a priori* based on WHO recommendations and priority areas for the health sector, we only assessed the quality of 10 indicators captured in the HMIS, limiting our ability to comment on representativeness of quality for the whole system. Finally, we did not assess reliability (consistency between paper registers at facilities) and accuracy (consistency between actual healthcare utilization at facilities and electronic reports) of Rwanda's HMIS data. Previous studies in Rwanda have looked at data reliability of the HMIS reports from community health workers as compared to register data. These studies found poor reliability of aggregated reports as compared to individual patient data (6). However, the bias was not systematically over- or under-reported and suggested that in aggregate, the errors might cancel out.

Our analysis demonstrates the feasibility of conducting a national assessment of HMIS data quality using the WHO data quality report card framework in a developing country. Since all of the indicators we studied are reported on a monthly basis to an electronic system, these methods can be replicated to provide routine monthly evaluations of HMIS completeness and internal consistency. We recommend maintaining and expanding these assessments for timely identification of HMIS data quality gaps and that all sub-Saharan African countries, including Rwanda, integrate these assessments into routine practice. We believe that routine assessments will lead to overall quality improvement of HMIS data and that this will encourage data use of this valuable system for program management and evaluation. We also hope these findings will allow other researchers to have more confidence in using these data for effective health sector decision-making.
